# Identification of *S23* causing both interspecific hybrid male sterility and environment-conditioned male sterility in rice

**DOI:** 10.1186/s12284-019-0271-4

**Published:** 2019-02-28

**Authors:** Chaowei Fang, Le Li, Runming He, Daiqi Wang, Man Wang, Qian Hu, Qianru Ma, Kaiyi Qin, Xueye Feng, Guiquan Zhang, Xuelin Fu, Ziqiang Liu

**Affiliations:** 1 State Key Laboratory for Conservation and Utilization of Subtropical Agro-Bioresources, South China Agricultural University, Guangzhou, 510642 China; 2 Guangdong Provincial Key Laboratory of Plant Molecular Breeding, College of Agriculture, South China Agricultural University, Guangzhou, 510642 China; 3 Present address: Huazhi Rice Bio-Tech Co., Ltd., Changsha, 410124 China; 4 Present address: Vegetable Research Institute, Guangdong Academy of Agricultural Sciences, Guangzhou, 510642 China

**Keywords:** *Oryza glumaepatula*, Chromosome single-segment substitution lines (SSSLs), Interspecific hybrid sterility, Environment-conditioned male sterility, *S23*

## Abstract

**Background:**

*Oryza glumaepatula* represents an important resource of genetic diversity that can be used to improve rice production. However, hybrid sterility severely restricts gene flow between *Oryza* species, and hinders the utilization of distant heterosis in hybrid rice breeding.

**Results:**

In order to fully exploit the beneficial genes of *O. glumaepatula* and facilitate the conservation of these gene resources, a set of chromosome single-segment substitution lines (SSSLs) was developed using an *indica* variety HJX74 as the recurrent parent and an accession of *O. glumaepatula* as the donor parent. During the process of SSSLs development, *S23*, a locus conferring hybrid male sterility between *O. sativa* and *O. glumaepatula*, was identified and fine mapped to 11.54 kb and 7.08 kb genomic region in *O. sativa* and *O. glumaepatula*, respectively, encoding three and two candidate ORFs, respectively. qRT-PCR and sequence analysis excluded one common *ORF* as the candidate gene. In addition, hybrid male sterility caused by *S23* was environment-sensitive, and could be observed only in natural short-day (NSD).

**Conclusion:**

Identification and candidate genes analysis of *S23* in this study provides a valuable example to study the crosstalk between interspecific F_1_ hybrid male sterility and environment-conditioned male sterility in rice, facilitates reserving and utilizing favorable genes or alleles of wild *Oryza* species, and allows for a more efficient exploitation of distant heterosis in hybrid rice breeding.

**Electronic supplementary material:**

The online version of this article (10.1186/s12284-019-0271-4) contains supplementary material, which is available to authorized users.

## Background

Agriculture relies heavily on the genetic diversity of crop plants. It is estimated that less than 15% of the potential diversity has been utilized in cultivated rice (http://www.fao.org/3/y4751e/y4751e0b.htm#bm11). The limited genetic diversity of cultivated rice renders them more vulnerable to environment and jeopardizes the potential for sustained genetic improvement over the long term (Tanksley and McCouch 1997). The wild species of the genus *Oryza* serve as a virtually untapped reservoir of genetic diversity that can be used to improve rice production (Brar and Khush 1997). Therefore, transferring genes that control desirable traits from wild rice to domestic cultivars has proven to be an important strategy in rice breeding (McCouch et al. 2007). Furthermore, hybrids from crosses between species have stronger hybrid vigor and greater yield potential than those within subspecies. However, hybrid sterility, a postzygotic reproductive barrier, is quite common in the hybrid plants which fail to produce fertile pollen or embryo sacs during reproductive development, thus restricting gene flow between *Oryza* species, maintaining species identity during speciation and hindering the utilization of distant heterosis in hybrid rice breeding (Ouyang and Zhang 2013).

Molecular genetic studies over two decades have revealed more than 40 loci causing interspecific or intersubspecific hybrid sterility in rice (Ouyang et al. 2009; Ouyang and Zhang 2013). Major progress has been made recently in understanding molecular mechanisms of hybrid sterility by molecular cloning of causal genes, revealing molecular mechanism of hybrid sterility in rice fitting two genetic models, one-locus sporo-gametophytic interaction model and the duplicate gametic-lethal model (Chen et al. 2008; Long et al. 2008; Yamagata et al. 2010; Mizuta et al. 2010; Yang et al. 2012; Kubo et al. 2016; Yu et al. 2016, 2018; Nguyen et al. 2017; Xie et al. 2017; Shen et al. 2017; Koide et al. 2018). The representative loci for the one-locus sporo-gametophytic model include hybrid female sterility loci *S5*, *S7* and *HSA1* (Chen et al. 2008; Yang et al. 2012; Kubo et al. 2016; Yu et al. 2016), and hybrid male sterility loci *Sa*, *Sc* and *qHMS7* (Long et al. 2008; Shen et al. 2017; Yu et al. 2018), as well as *S1* which confers both hybrid male and female sterility (Xie et al. 2017; Koide et al. 2018). Each locus contains two or more closely located genes interacting to cause gamete sterility, such as three genes at *S5* locus functioning in “Killer-Protector” model (Yang et al. 2012), two genes at *Sa* locus functioning in “two gene/three component interaction” model (Long et al. 2008), two genes in *qHMS7* functioning in “Toxin-Antidote” model (Yu et al. 2018), multiple alleles at *Sc* locus functioning in allelic suppression pattern (Shen et al. 2017), and two genes in *HSA1* locus functioning in an allele-specific epistatic interaction pattern (Kubo et al. 2016). Although *S1* was identified as a single gene, different groups revealed different causal gene responsible for *S1*-induced hybrid sterility, suggesting a more complicated molecular mechanism for *S1* instead of a simple model of a single gene function (Xie et al. 2017; Koide et al. 2018). In the duplicate gametic-lethal model, gamete-essential genes were duplicated on different chromosomes, followed by reciprocal loss of one of the duplicated genes in the divergent species. Therefore, in the hybrids, genetic segregation and recombination gave rise to gametes with none of the duplicated functional genes, leading to hybrid sterility. In rice, reciprocal loss of duplicated gene pairs, such as *S27*/*S28*, *DPL1*/*DPL2* and *DSG1/DSG2*, produces about 25% sterile pollen grains that lack both of the functional loci in hybrids, thus contributed to reproductive barrier in interspecific or intersubspecific crosses (Yamagata et al. 2010; Mizuta et al. 2010; Nguyen et al. 2017).

In order to fully exploit the beneficial genes of wild *Oryza* species and facilitate the conservation of these gene resources, we started to develop SSSLs in the genetic background of an *indica* cultivar HJX74 using accessions from wild *Oryza* species as donors, and had developed 99 SSSLs carrying donor segments of *O. meridionalis* (He et al. 2017). In the present study, we identified a locus *S23* conferring environment-sensitive hybrid male sterility between *O. sativa* and *O. glumaepatula*, another wild *Oryza* species belonging to AA genome group, during the process of the development of SSSLs using an accession of *O. glumaepatula* as the donor parent. *S23* was fine mapped to 11.54 kb and 7.08 kb genomic region in *O. sativa* and *O. glumaepatula*, respectively, encoding three and two candidate ORFs, respectively. Candidate genes were analyzed and their interaction with environment was discussed. Based on these findings, *S23* can be selected as a target for genome editing to break down the reproductive barrier between cultivated rice and wild rice for reserving and utilizing favorable genes or alleles of wild *Oryza* species, and for facilitating hybrid rice breeding.

## Results

### A segregation distortion observed in SSSLs development

**Fig. 1:**
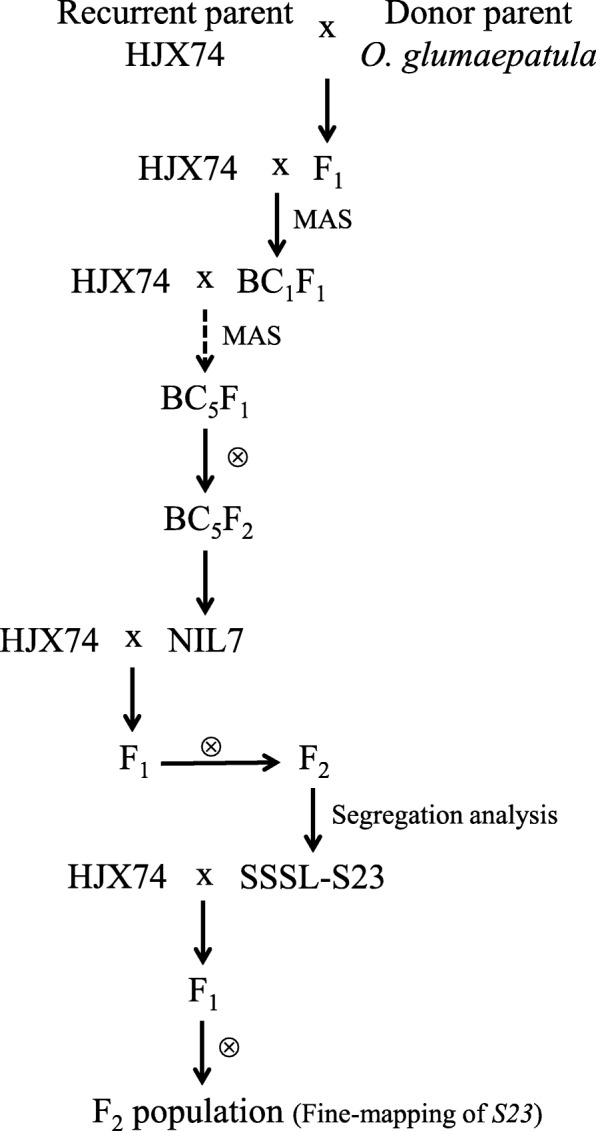
Flowchart of the development and analysis of SSSL-S23. MAS: marker-assisted selection

The procedure for the development and analysis of SSSL was summarized in Fig. 1. Totally, 168 SSSLs were developed, with the substituted segments covering 81% of the rice genome (Unpublished data).

In BC_5_F_2_ generation, NIL7 was detected to have two substituted segments on chromosome 7 and chromosome 12, respectively (Fig. 1 and Additional file 1: Figure S1). Therefore, NIL7 was crossed with HJX74 in order to develop two SSSLs with only one substituted segment for each SSSL. Under NSD condition, two hundred and twenty-one F_2_ plants were genotyped using 5 SSR (Simple Sequence Repeats) markers (RM47, M429, M22060, PSM148 and M22172) on the substituted segment of chromosome 7 and 2 SSR markers (RM17, MM2968) on the substituted segment of chromosome 12. A segregation distortion of the genotype was found for the 5 SSR markers on chromosome 7. The homozygous genotype for *O. glumaepatula* was significantly lower than the expected 25%, suggesting that the gametes (male or female) derived from *O. glumaepatula* were selectively aborted and were not transmissible to the progeny. In contrast, the segregation of the 2 SSR markers on chromosome 12 was normal, fitting the 1:2:1 ratio (Table 1). Our results indicated the substituted segment on chromosome 7 contained a gene locus controlling hybrid sterility between *O. sativa* and *O. glumaepatula*.

**Table 1:** Segregation analysis of the F_2_ families derived from crossing NIL7 with HJX74

Chromosome	Molecular marker	No. of F_2_ plants			χ^2^(1:2:1)	*P* value
		H/H	H/G	G/G		
7	RM47	85	113	13	50.20	5.51E-15***
	M429	86	118	7	67.39	3.24E-14***
	M22060	89	116	6	62.12	2.33E-15***
	PSM148	90	109	12	57.90	2.67E-13***
	M22172	81	109	20	35.58	1.73E-08***
12	RM17	54	97	58	1.24	0.541
	MM2968	53	97	59	1.43	0.491

Note: H/H, G/G and H/G represented the homozygous genotype for HJX74, *O. gulmaepatula* and the heterozygous genotype, respectively, at the corresponding molecular markers. *P* value was determined with a Student’s *t*-test analysis. *** represented significance at *P* = 0.001

### Identification of *S23* conferring hybrid male sterility

**Fig. 2:**
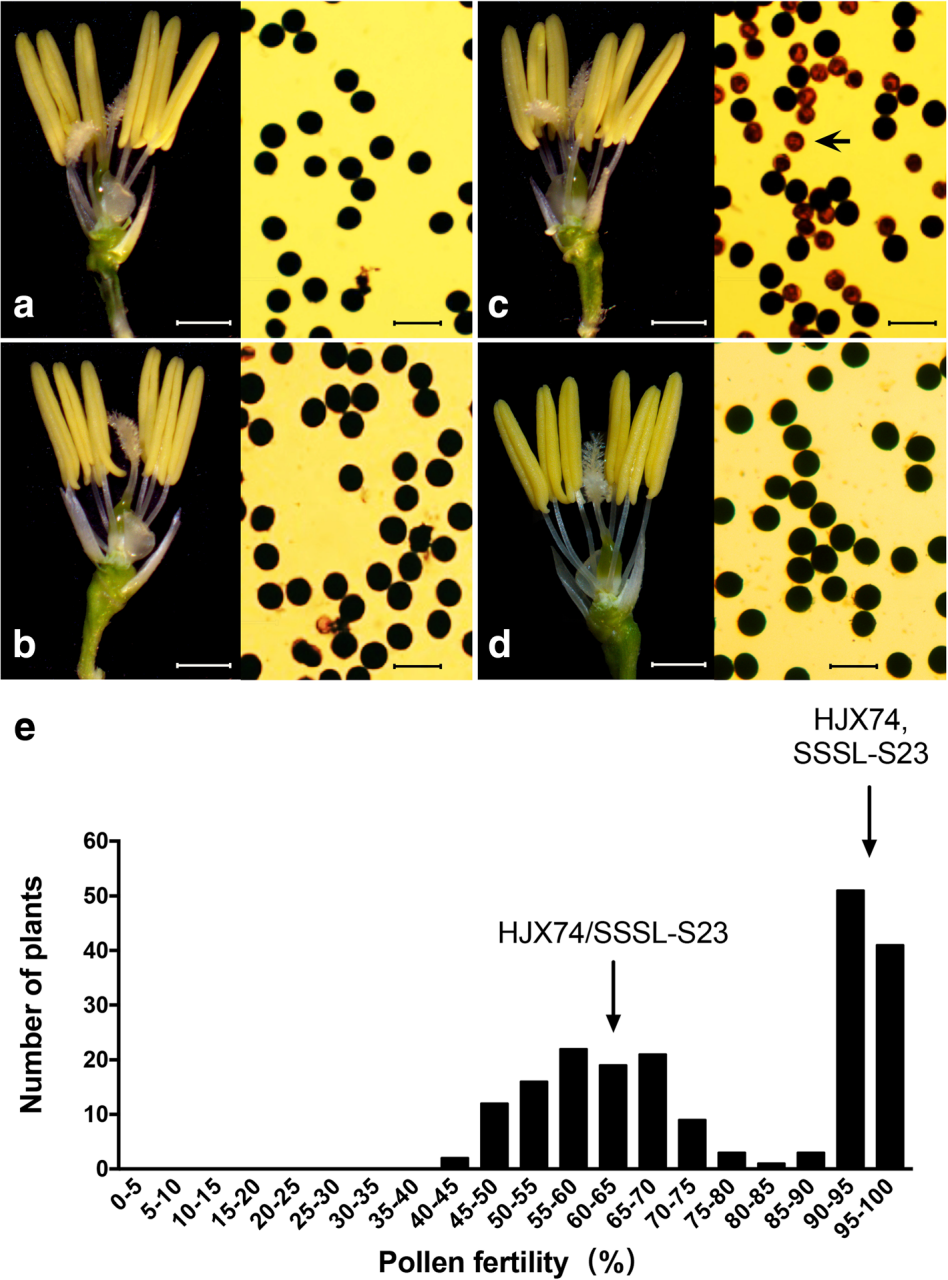
Pollen fertility of HJX74, SSSL-S23, HJX74/SSSL-S23 F_1_ and F_2_ populations. Spikelets (left) and pollen grains (right) in HJX74 (**a** in NSD), SSSL-S23 (**b** in NSD) and HJX74/SSSL-S23 F_1_ (**c** in NSD and **d** in NLD) plants. Arrow indicated the aborted pollen grain. Scale bars, 1 mm for spikelet and 100 μm for pollen. **e** A bimodal distribution for pollen fertility detected in 200 F_2_ plants derived from the cross between HJX74 and SSSL-S23 in NSD. Arrows indicated the mean pollen fertility of HJX74, SSSL-S23 and HJX74/SSSL-S23 F_1_ plants, respectively

SSSL-S23, a SSSL with only the substituted segment on chromosome 7 selected from the F_2_ population generated from crossing NIL7 with HJX74, was further crossed with HJX74 to generate F_1_ and F_2_ population for analyzing hybrid sterility locus (Fig. 1). Under NSD condition, the pollen stainability conducted between the parents and the F_1_ hybrid under microscope showed that the pollen fertility of parents HJX74 and SSSL-S23 were normal (Table 2, Fig. 2a and b), whereas their F_1_ hybrid showed clear pollen sterility (Table 2 and Fig. 2c), and the type of pollen abortion was stained abortion (Fig. 2c). Although the pollen sterility was observed, the anther developed normal in F_1_ hybrid (Fig. 2a-c). The spikelet fertility of F_1_ hybrid was 89.61 ± 4.34%, similar to those of the parents (Table 2), indicating that the embryo sac of the F_1_ hybrid was normal.

**Table 2:** Pollen and spikelet fertility of the parents and F_1_ hybrid

Growth condition	Materials	Pollen fertility (%)	Spikelet fertility (%)
NSD	HJX74	94.95 ± 5.24 A ^a^	92.63 ± 4.31 A
	SSSL-S23	95.23 ± 3.29 A	90.21 ± 4.98 A
	HJX74/SSSL-S23	63.15 ± 13.49 B	89.61 ± 4.34 A
NLD	HJX74	93.91 ± 1.94 A	89.16 ± 4.24 A
	SSSL-S23	94.48 ± 2.14 A	88.37 ± 4.89 A
	HJX74/SSSL-S23	92.99 ± 2.38 A	91.78 ± 4.42 A

Note: Pollen fertility and spikelet fertility were shown as mean ± SD. ^a^ Duncan’s multiple comparison test was conducted for pollen fertility and spikelet fertility. Numbers followed by different letters in each row represented statistically significant difference at *P* = 0.01, while numbers with the same letter indicated no significant difference at *P* = 0.05

The distribution of pollen sterility in 200 HJX74/SSSL-S23 F_2_ plants showed a clear bimodal pattern with an apparent valley at 80–90% in NSD (Fig. 2e). Taking 80–90% as the cut-off region between sterility and fertility, the population segregated for sterility (< 80%): fertility (> 90%) at the ratio of 1:1 (χ^2^ = 0.8 < χ^2^_0.05,1_ = 3.84), demonstrating monogenic inheritance for the trait. We concluded that hybrid male sterility in our population was determined by a single gene on the substituted segment of chromosome 7 (named as *S23*). Interestingly, the pollen sterility of F_1_ hybrid could only be observed in NSD (Table 2 and Fig. 2c), but not in natural long-day (NLD) (Table 2 and Fig. 2d), suggesting the function of *S23* was environment-conditioned, and was possibly regulated by photoperiod and/or temperature.

**Fig. 3:**
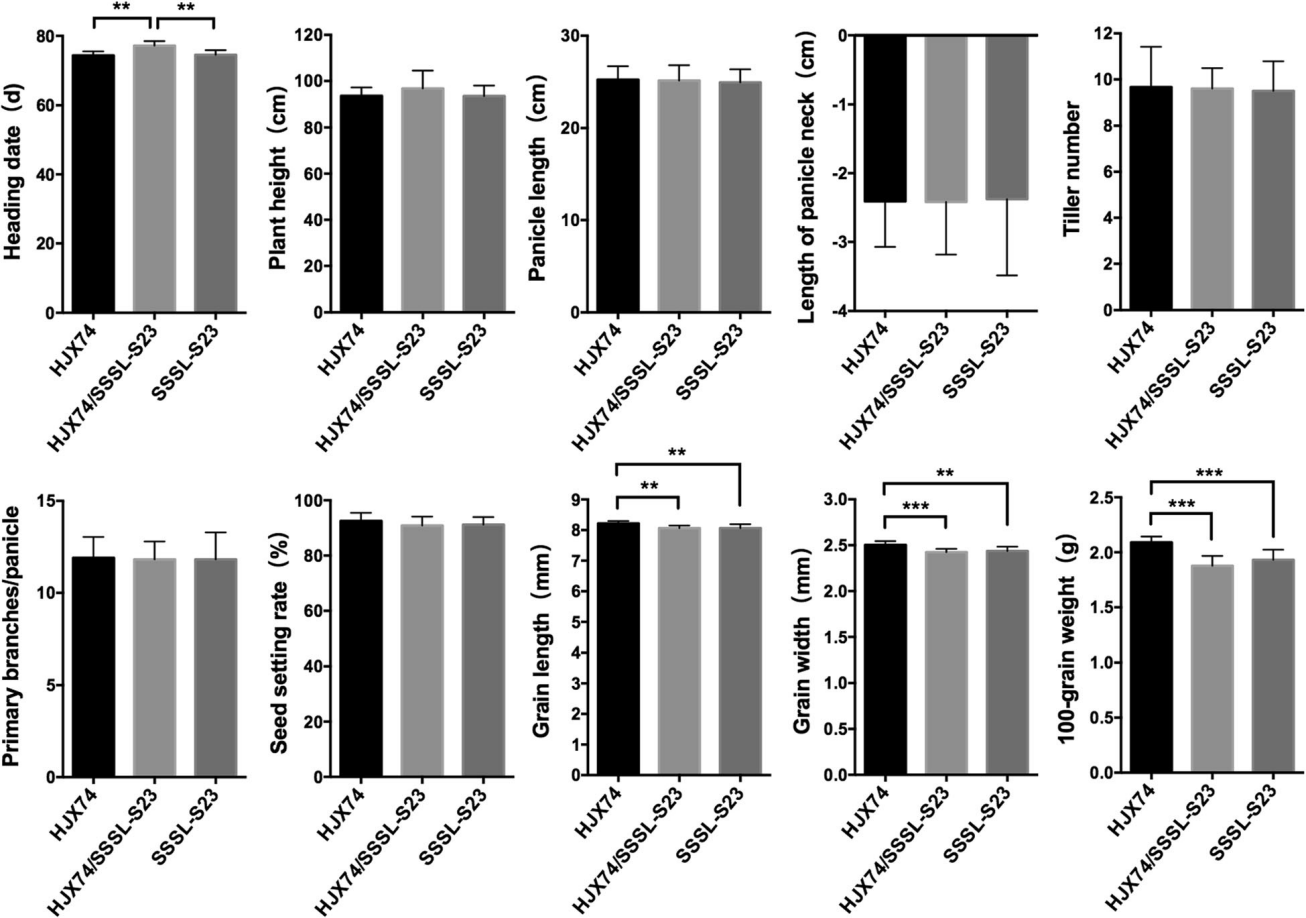
Ten key agronomic traits in HJX74, SSSL-S23 and HJX74/SSSL-S23 F_1_ plants in NSD. ** and *** represented significance at *P* = 0.01 and *P* = 0.001, respectively

The agronomic traits of HJX74, SSSL-S23 and their F_1_ hybrid were also evaluated in NSD (Fig. 3). No significant differences were found for plant height, panicle length, panicle exertion, number of tillers per plant, number of primary branches per panicle and seed setting rate among the three genotypes (Fig. 3). However, heading of F_1_ hybrid was significantly delayed compared to the two parents, suggesting that *S23* might also have effect on heading date. The grain length, grain width and 100-grain weight were significantly lower in F_1_ hybrid and SSSL-S23 compared with HJX74 (Fig. 3), suggesting that the substitution segment on chromosome 7 from wild rice might have a dominant QTL conferring small grain phenotype.

### Fine mapping of *S23*

**Fig. 4:**
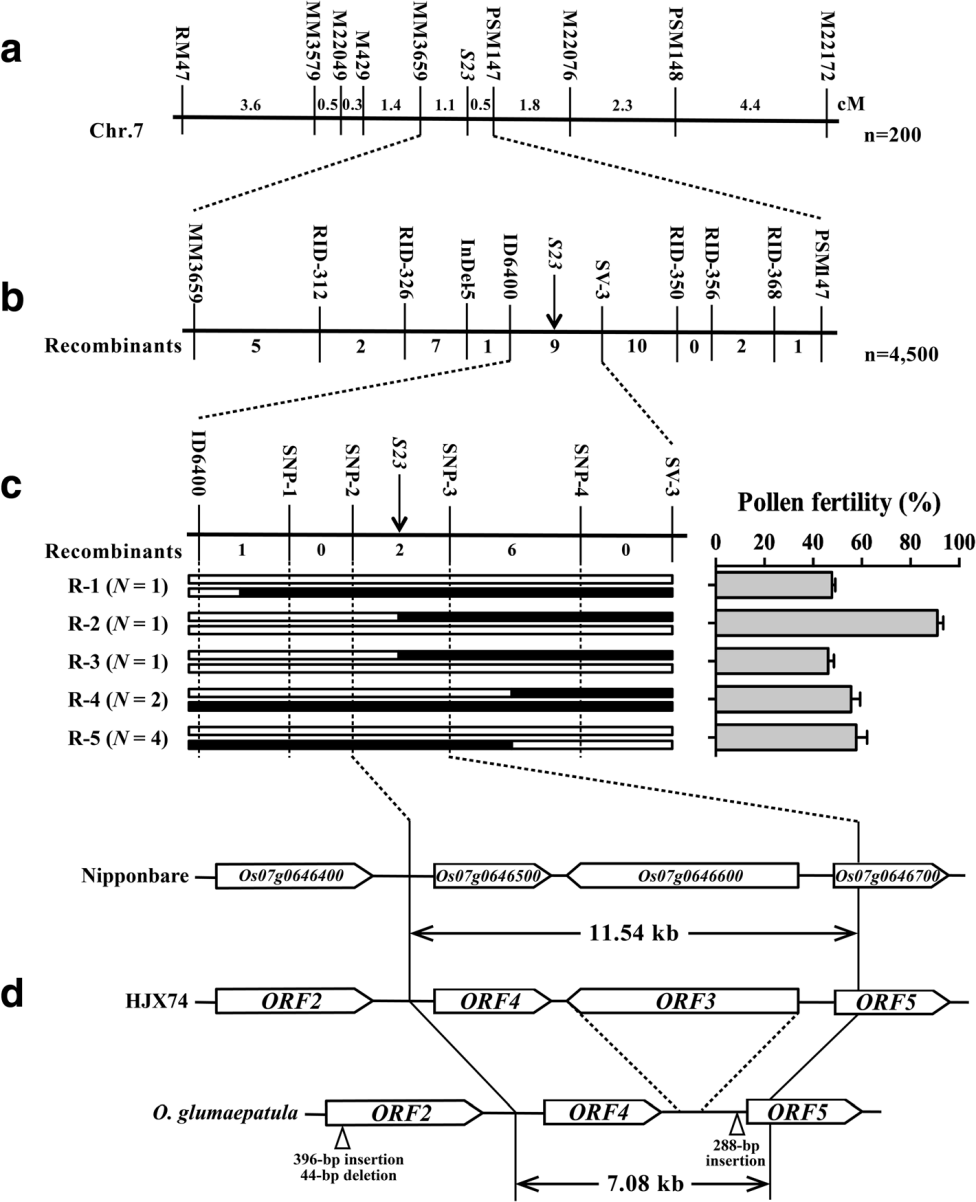
Map-based cloning of *S23*. (a and b) *S23* was mapped to a 1.6 cM region flanked by MM3659 and PSM147 using 200 HJX74/SSSL-S23 F_2_ plants (**a**), and was then fine-mapped to the region between ID6400 and SV-3 using 4500 HJX74/SSSL-S23 F_3_ plants (**b**). **c** Nine key recombinants defined *S23* to the region between SNP-2 and SNP-3. In Nipponbare genome, there were three genes in the 11.54 kb target region between SNP-2 and SNP-3. White and black boxes indicated chromosomal segments from HJX74 and *O. glumaepatula*, respectively. Genetic distance and the number of recombinants between adjacent markers was shown above and below the bar, respectively. **d** Sequence analysis was performed for the fine-mapped region and neighbouring region of HJX74 and *O. glumaepatula*, and *ORFs* were predicted

Under NSD condition, two hundred HJX74/SSSL-S23 F_2_ plants were used to analyze the linkage between pollen fertility and SSR markers, and *S23* locus was pinpointed to an interval between MM3659 and PSM147 (Fig. 4a). Twelve new markers were developed in the marker interval of MM3659-PSM147, and were used to analyze a total of 4500 F_3_ plants. As a result, nine recombinants were identified between markers ID6400 and SV-3 (Fig. 4b). The self-pollinated progenies (F_4_ lines) of those nine plants were used to determine the genotypes of *S23* (Fig. 4c). Based on the sequence of a *japonica* standard, Nipponbare, the recombinants R-2 and R-3 restricted *S23* to 11.54 kb genomic region between the SNP-2 and SNP-3 markers, including three putative genes (*Os07g0646500*, *Os07g0646600* and *Os07g0646700*, named as *ORF4*, *ORF3* and *ORF5*, respectively) (Fig. 4c). Sequence analysis revealed that there were also three genes (*ORF3*^*HJX74*^, *ORF4*^*HJX74*^ and *ORF5*^*HJX74*^) in the 11.54 kb *S23* region in HJX74, but only two genes (*ORF4*^*Glu*^ and *ORF5*^*Glu*^) in the 7.08 kb *S23* region in SSSL-S23 (Fig. 4d). Both *ORF4* and *ORF5* are predicted to encode a SWIM-type zinc finger and MULE transposase domain-containing protein, and *ORF3* is predicted to encode a 496-aa protein without any conserved domains. Sequence comparation of the candidate genes in HJX74 and SSSL-S23 revealed nucleotide diversity for *ORF5* but not for *ORF4*. Four SNPs were found in the coding region of *ORF5* between HJX74 and SSSL-S23, resulting in two amino acid substitutions (Additional file 1: Figure S2). Interestingly, a 288-bp deletion was found in *ORF5*^*HJX74*^ promoter region compared to that of *ORF5*^*Glu*^ (Fig. 4d). Indel (Insertion and deletion) was also found in *Os07g0646400* (named as *ORF2*) which located just outside of the candidate mapping region of *S23*. Compared to the sequence of *ORF2*^*HJX74*^, a 398-bp insertion, a 44-bp deletion and several SNPs were found in *ORF2*^*Glu*^, resulting in sequence difference of a 24-residue insertion/deletion and eight amino acid substitutions for the encoded proteins (Fig. 4d and Additional file 1: Figure S3a).

### Candidate gene analysis of *S23*

**Fig. 5:**
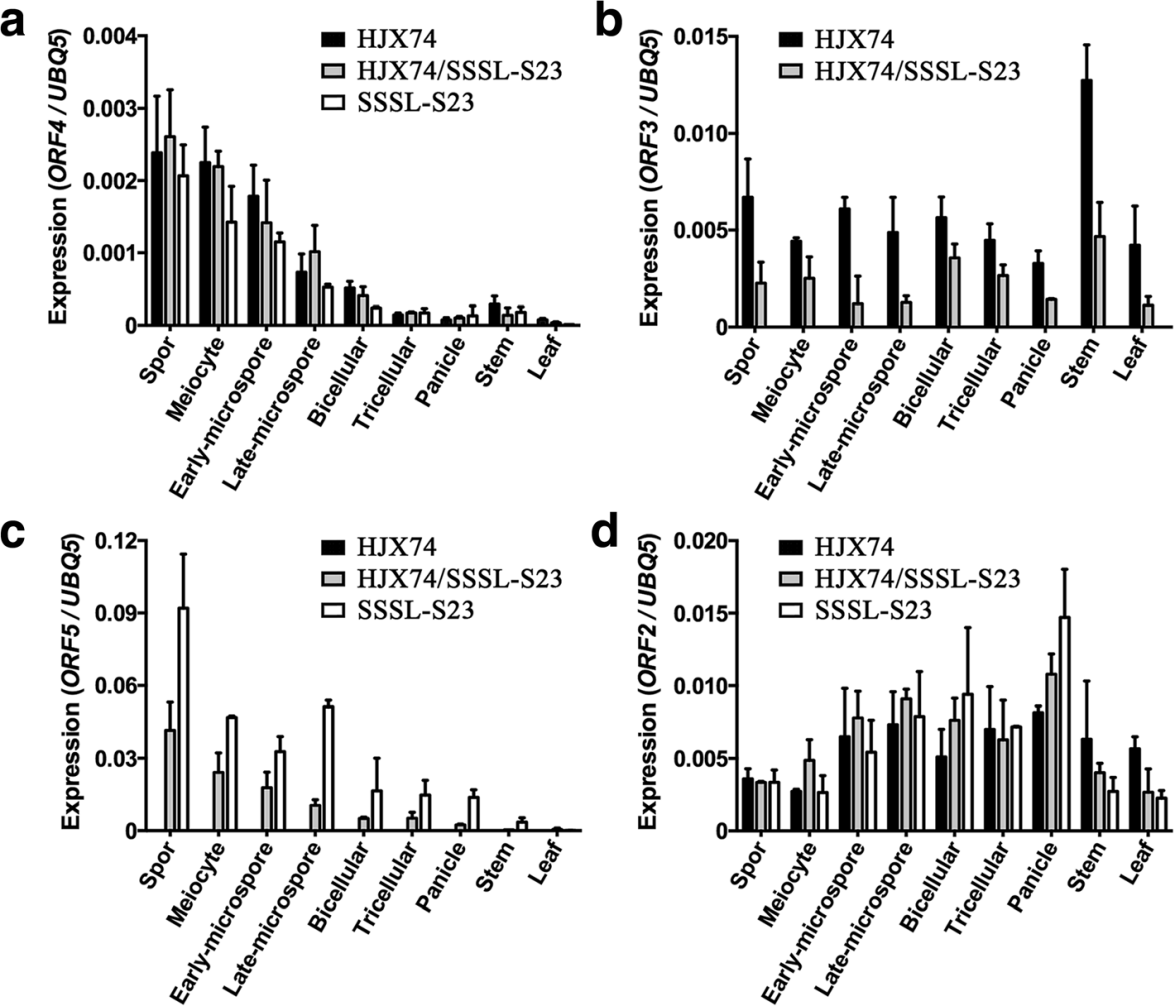
Expression analysis of *S23* candidate genes. The expression of *ORF4* (**a**), *ORF3* (**b**), *ORF5* (**c**) and *ORF2* (**d**) was analyzed in developing anthers from sporogenous cell stage to tricellular pollen stage and in other tissues including panicles, stems and leaves of HJX74, SSSL-S23 and HJX74/SSSL-S23 F_1_ plants in NSD. Spor, sporogenous cell stage

To define candidate genes for *S23*, we examined the expression of the three target genes as well as *ORF2* in which major sequence differences were found between the two parent genotypes in NSD. All genes were highly expressed in different stages of anther development, but only *ORF3* and *ORF2* showed expression in vegetative organs (Fig. 5). *ORF4* was equally expressed among HJX74, SSSL-S23 and F_1_ hybrid (Fig. 5a). In combination with the consideration that *ORF4* sequence was identical between HJX74 and SSSL-S23, we speculated that *ORF4* was not the candidate gene for *S23*. *ORF3* was stably expressed during anther development, while *ORF5* expression was slightly higher in early stages of anther development, and was gradually reduced in later stages (Fig. 5b and c). As expected, no expression of *ORF3* was detected in SSSL-S23 since *ORF3* was absent in SSSL-S23 (Fig. 5b). Interestingly, *ORF5* was expressed in SSSL-S23, but not in HJX74 (Fig. 5c), suggesting that the 288-bp deletion in HJX74 completely disrupted the expression of *ORF5*. In F_1_ hybrid, the expression of *ORF3* and *ORF5* was about half of that in HJX74 and SSSL-S23, respectively (Fig. 5b and c), in consistent with that only one allele of *ORF3* and *ORF5* was expressed in F_1_ hybrid. The expression of *ORF2* was lower in early stages of anther development, and was gradually increased in later stages (Fig. 5d). Furthermore, *ORF2* was equally expressed among HJX74, SSSL-S23 and F_1_ hybrid (Fig. 5d), suggesting the major sequence differences identified in *ORF2* possibly interfered with the protein function instead of affecting its transcription.

## Discussion

The wild species of *Oryza* are an important reservoir of useful genes. Numerous genes conferring resistance or tolerance to biotic and abiotic stresses have been identified in wild species of *Oryza* (Brar and Khush 1997). Although the overall economical characters of wild rice are inferior to those of cultivated rice, previous reports demonstrated that many favorable genes hidden in wild rice are essential for yield-related trait improvement (Xiao et al. 1996, 1998; Tian et al. 2006; Huang et al. 2012). However, hybrid sterility, which is the most common form of postzygotic isolation and plays an important role in maintaining species identity (Orr and Presgraves 2000), is the major obstacle for the utilization of *O. glumaepatula* specific traits in improving rice varieties. Until now, only a few pollen eliminators have been identified in the hybrid between *O. sativa* and *O. glumaepatula*, and only *S27/S28* gene pairs had been molecularly analyzed (Sano 1994; Sobrizal et al. 2000a, b, Sobrizal and Yoshimura, 2001, 2002; Yamagata et al. 2010; Sakata et al. 2014; Zhang et al. 2018). In order to identify and transfer favorable alleles from *O. glumaepatula* into cultivated rice varieties, and to facilitate their application in rice breeding, a set of SSSLs was developed in the genetic background of an elite *indica* cultivar HJX74 using wild rice *O. glumaepatula* as the donor parent. During the process of SSSLs development, an interspecific hybrid sterile locus *S23* was identified to be involved in male gamete development in hybrids between *O. sativa* and *O. glumaepatula*, and was then fine mapped to 11.54 kb and 7.08 kb genomic region in *O. sativa* and *O. glumaepatula*, respectively, encoding three and two candidate ORFs, respectively. *S23* was first identified in 2000 during the development of *O. glumaepatula* introgression lines in the background of *O. sativa* cv. Taichuang 65, and it was found to be co-segregated with RFLP marker C1340 (Sobrizal et al. 2000a), which was close to the position of *S21* from *O. glaberrima* and *O. rufipogon* for hybrid male sterility (Doi et al. 1999; Miyazaki et al. 2006). Recently, *qHMS7*, which conferred hybrid male sterility between *O. meridionalis* and *O. sativa*, was cloned and contained two tightly linked genes (*ORF2* and *ORF3*) (Yu et al. 2018). *ORF2*, which was functional in *O. sativa* but was non-functional in *O. meridionalis*, encoded a toxin affecting the development of pollen, and *ORF3*, which was present in *O. sativa* but was missing in *O. meridionalis*, encoded an antidote required for pollen viability. Mer-type pollens lacking *ORF3* were selectively eliminated, leading to segregation distortion in the progeny. Sequence analysis revealed that *S23* was allelic with *qHMS7*, and *S23-ORF3* was identical to *qHMS7-ORF3* (Additional file 1: Figure S3b). Interestingly, *qHMS7-ORF2* didn’t locate in the mapping region of *S23*, since the recombinants R-2 and R-3 restricted *S23* to the genomic region between the SNP-2 and SNP-3 markers (Fig. 4c). However, sequencing of the recombinants R-2 and R-3 revealed that both recombinants fitted the “Toxin-Antidote” model of *qHMS7* as they both contained *ORF2* allele from *O. sativa*, while one and two alleles of *ORF3* existed in R-3 and R-2, respectively (Additional file 1: Figure S4). Therefore, when map-based cloning QTLs of hybrid sterility, neighboring regions of the target site should be also analyzed even if the result of the recombinants has suggested otherwise.

Although *S23* was allelic to *qHMS7*, the genetic effect was not completely the same. F_1_ pollen fertility was 51.16 ± 1.29% for *qHMS7*. Gametophytic transmission analysis using the backcross population of NIL-*qHMS7*/DJY1 and the self-pollinated population of NIL-*qHMS7* identified no heterozygous genotype and no homozygote from *O. meridionalis* at *qHMS7* locus, respectively, indicating the pollen grains carrying *O. meridionalis* allele aborted completely (Yu et al. 2018). However, F_1_ pollen fertility was 63.15 ± 13.49% for *S23* (Table 2), and homozygote from *O. glumaepatula* at *S23* locus could be identified (Table 1), indicating that the pollen grains carrying *O. glumaepatula* allele was not completely sterile. We speculated that the “Toxin” in our study was not as toxic as that in *qHMS7* research, or the “Antidote” in *S23* locus was super effective. Since *ORF2* was equally expressed among HJX74, SSSL-S23 and F_1_ hybrid (Fig. 5d), a possible explanation of the phenotypic difference might be the different ORF2 function due to sequence variation. Although *qHMS7-ORF2* was sequenced in 89 accessions of wild *Oryza* species, 210 accessions of *O. sativa* and 11 accessions of *O. glaberrima* collected from 29 countries and regions over diverse geographical area, and 27 haplotypes were identified among the sequenced accessions, *ORF2* sequence in *O. glumaepatula* was untouched (Yu et al. 2018). Our sequence analysis revealed a 24-residue insertion/deletion and eight amino acid substitutions between ORF2^Glu^ and ORF2^HJX74^ (Additional file 1: Figure S3a). However, the sequence of functional S23-ORF2^HJX74^ and qHMS7-ORF2^D^ was identical (Additional file 1: Figure S3a), indicating the toxin function of S23-ORF2^HJX74^ was not affected compared with qHMS7-ORF2^D^. Furthermore, ORF3 sequence was completely same between ORF3^D^ in *qHMS7* and ORF3^HJX74^ in *S23* (Additional file 1: Figure S3b), and the expression of *ORF3*^*HJX74*^ was unaffected (Fig. 5b), indicating that the efficacy of the antidote S23-ORF3^HJX74^ might not be the reason for the different genetic effect. Therefore, an unidentified partner should play some different role in *S23* and *qHMS7*. A possible candidate was *ORF1*, which was homologous to *ORF3* and located just next to *ORF2* (Yu et al. 2018). Several polymorphic sites near the C-terminal region of ORF1 compared with ORF3 might affect the protein function (Additional file 1: Figure S3b). Furthermore, transformation of intact *ORF1*^*D*^ genomic sequence into the hybrid D/M-type plants could not restore the pollen fertility, suggesting that ORF1^D^ was non-functional (Yu et al. 2018). However, the expression of *ORF1*^*HJX74*^, which was supposed to similar to the *O. sativa* allele *ORF1*^*D*^, was nearly undetectable, while the expression of *ORF1*^*Glu*^ reached about one-tenth to one-fifth expression level of *ORF3* (Fig. 5b and Additional file 1: Figure S5). Therefore, trace level of *ORF1*^*Glu*^ could possibly partially restored the pollen fertility in HJX74/SSSL-S23 F_1_ hybrid plants. *ORF5* could also be a possible candidate. A 288-bp deletion were found in *ORF5*^*HJX74*^ promoter region compared to that of *ORF5*^*Glu*^ (Fig. 4d), and completely disrupted the expression of *ORF5*^*HJX74*^. Whether ORF5^Glu^ performs an antidote-like function to alleviate the toxicity of ORF2^HJX74^ remains to be investigated.

Another phenotypic difference caused by *S23* and *qHMS7* was that the pollen sterility of F_1_ hybrid in our study could only be observed in NSD, but not in NLD (Table 2, Fig. 2c and d). Fertile pollen in F_1_ hybrid of HJX74 and SSSL-S23 had been observed in the NLD of four consecutive years in Guangzhou, China, suggesting the function of *S23* was environment-conditioned. Since ORF2^HJX74^ in *S23* was identical to ORF2^D^ in *qHMS7*, we assumed that the expression of *ORF2*^*HJX74*^ might be regulated by photoperiod and/or temperature, resulting in the absence or low level of toxin in NLD. The trait of photoperiod- and/or thermo-sensitive male fertility-sterility conversion had been widely used in two-line system in hybrid rice breeding, and the underlying mechanisms of some acting genes and lncRNAs had been recently studied (Zhou et al. 2012, 2014; Ding et al. 2012; Zhang et al. 2013; Fan et al. 2016; Fan and Zhang 2018). However, none of them had been reported to be involved in hybrid sterility. To our knowledge, this is the first report on a single locus controlling both hybrid sterility and environment-sensitive sterility. Thus, our findings provided a valuable example of *S23* to study the crosstalk between F_1_ hybrid male sterility and environment-conditioned male sterility in rice.

*S23* causing male gamete abortion in the heterozygous condition limits the utilization of favorable genes not only in *O. glumaepatula*, but also in other wild *Oryza* species. Understanding the nature of this gene offers approaches to overcome male sterility in wild rice-cultivated rice hybrids, thus facilitating utilization of the strong hybrid vigor. *O. sativa* carrying non-functional *S23-ORF2* can be selected as wide-compatible lines to break down the reproductive barrier and overcome hybrid sterility in the interspecific hybrid breeding. Furthermore, the genome editing-based approach can rapidly generate neutral alleles at *S23* by creating loss-of-function *S23-ORF2*, allowing broader and easier access to desirable traits in distantly related species.

## Conclusions

A set of SSSLs was developed in the genetic background of an elite *indica* cultivar HJX74 using wild rice *O. glumaepatula* as the donor parent, and *S23* was identified to cause both interspecific hybrid male sterility and environment-conditioned male sterility in rice. Identification and candidate genes analysis of *S23* in this study provides a valuable example to study the crosstalk between interspecific F_1_ hybrid male sterility and environment-conditioned male sterility in rice, facilitates reserving and utilizing favorable genes or alleles of wild *Oryza* species, and allows for a more efficient exploitation of distant heterosis in hybrid rice breeding.

## Methods

### Plant materials and growth conditions

To develop SSSLs, the recipient parent HJX74, an elite *indica* variety from south China, was crossed with the donor parent wild rice *O. glumaepatula* (accession number IRGC104387), and the F_1_ plants were backcrossed with HJX74 to develop the BC_1_F_1_ generation. Polymorphic SSR markers were used in the selection of the donor chromosomal segments. Using the same method, BC_5_F_1_ plants were obtained, and were self-crossed to produce BC_5_F_2_ lines in which the majority of genomic regions were homozygous for HJX74 alleles. 191 polymorphic markers evenly distributed over 12 chromosomes were selected to detect the chromosome substituted segments. SSSL which had only one substituted segment from the wild rice *O. glumaepatula* could be obtained in this generation. Plants with two or more substituted segments would be crossed with HJX74 again to produce more SSSLs.

Plants were grown under NSD (from July to November) and NLD (from March to July) conditions in a paddy field in Guangzhou (23°07′N, 113°15′E), China. In NSD, the average temperature in July, August, September, October and November is approximately 27.9 °C, 28.7 °C, 27.9 °C, 23.6 °C and 19.3 °C, respectively, while in NLD, the average temperature in March, April, May, June and July is approximately 17.9 °C, 21.6 °C, 25.1 °C, 28.2 °C and 27.9 °C, respectively.

### PCR analysis and development of molecular markers

Fresh leaves were collected at the seedling stage and then ground in liquid nitrogen. Microquantities of DNA were extracted from fresh leaves of each individual using a previously reported method (Chen et al. 2014). Amplification was carried out on the program for the initial denaturing step with 94 °C for 3 min, followed by 35 cycles for 30 s at 94 °C, 30 s at 55 °C, 30 s at 72 °C, with a final extension at 72 °C for 5 min. PCR products were separated on 6% nondenaturing polyacrylamide gel and detected using the silver staining method. SSR markers were selected covering the target region based on the published linkage map of rice (http://www.gramene.org). Indel and SNP markers used for fine mapping of *S23* were designed based on the result of the sequence analysis of HJX74 and SSSL-S23.

### Examination of pollen and spikelet fertility

The examination of pollen and spikelet fertility was as described previously (Guo et al. 2016). Briefly, to examine pollen fertility, 6–9 mature flowers were collected from the upper one-third of the panicles of plants during the flowering time and fixed in FAA solution (ethanol, formaldehyde and acetic acid at a ratio of 89:6:5). The pollen was stained with 1% I_2_-KI solution containing 0.1% (*w*/*v*) iodine and 1% (w/v) potassium iodide. More than 300 pollen grains were randomly scanned per plant. The pollen could be divided into two types: normal pollen (normal size and fully stained) and stained abortive pollen (small size and lightly stained). Pollen fertility was estimated by the proportion of fertile pollen grains present. To examine spikelet fertility, three main panicles per plant were harvested and calculated for the mean seed set. Ten to twenty plants were recorded for each variation.

### Agronomic traits evaluation

Agronomic traits were evaluated under NSD condition. Heading date was defined as the time when the first panicle emerged. Plant height and number of tillers per plant was measured at maturity stage and tilling stage, respectively. Yield-related traits including panicle length, panicle exertion, number of primary branches per panicle, seed-setting rate, grain length, grain width and 100-grain weight were investigated after rice harvested at maturity stage and sun dried. Panicle exertion was calculated as the distance between the leaf cushion of flag and the neck-panicle node. Grain length and grain width were determined by using Microtek ScanWizard EZ scanner and SC-E Image analysis software (Hangzhou Wanshen Detection Technology Co., Ltd., Hangzhou, China). Ten to twenty plants were recorded for each variation. Differences among the three genotypes were determined using one-way ANOVA.

### Sequence analysis

A number of primer pairs were designed based on the reference Nipponbare genomic sequence and long-length PCR with KOD Plus Neo DNA polymerase (TOYOBO, Japan) was used to amplify the *S23* region sequences from gDNAs of HJX74 and SSSL-S23 in multiple fragments. The PCR products were then sequenced. The sequences were assembled using SeqMan of the Lasergene package, and analyzed by BLAST in VectorNTI package.

### RNA isolation, RACE and qRT-PCR analysis

Total RNAs from rice tissues (anthers, young panicles, leaves and stems) of HJX74, SSSL-S23 and their hybrid were isolated using TRIZOL reagent (Invitrogen) following the manufacturer’s instruction. First-strand cDNA was reverse transcribed from DNaseI-treated RNA with oligo-dT as the primer using ReverTra Ace kit (Toyobo). For the RACE assay, the full-length transcripts were amplified by nested PCR with the SMARTer RACE cDNA Amplification kit (Clontech). Gene expression was measured by qRT-PCR using the ABI 7500 system (Life technologies). The qRT-PCR was carried out in a total volume of 20 μl containing 1× SYBR Green Master Mix (Life technologies). We normalized the expression levels by using UBQ5 gene as internal control. Each set of experiments was repeated three times, and the relative standard curve quantification method was used to evaluate quantitative variation. The qRT-PCR procedure was conducted at 94 °C for 3 min, followed by 40 cycles at 94 °C for 30 s, 58 °C for 30 s, and 72 °C for 30 s. The primers used were listed in Additional file 2: Table S1.

## Additional files

Additional file 1: **Figure S1.** Graphic genotype of NIL7. Black bars indicated the genomic fragments from *O. glumaepatula* and the other parts were from HJX74. **Figure S2.** Alignment of the deduced amino acid sequence of ORF5^Glu^ and ORF5^HJX74^. The two amino acid substitutions were shown in red. **Figure S3.** Haplotype analysis of ORF2 and ORF1/3. The gene structures of *ORF2* (a) and *ORF1/ORF3* (b) were shown on the top and on the bottom, respectively. The black and grey blocks indicated translated regions and untranslated regions, respectively. The variable sites were shown by the vertical lines. The site numbers indicated the positions of amino acid in ORF2^HJX74^/ORF2^D^ and ORF3 for the respective polymorphic sites, respectively. The haplotype numbers in parentheses indicated the length of the deduced proteins for the respective haplotypes. ORF2^HJX74^/ORF2^D^ and ORF3 were functional, while ORF2^Glu^, ORF2^M^ and different haplotypes of ORF1 were supposed to be non-functional. The polymorphic sites in ORF2^Glu^ compared with ORF2^M^, in ORF1^HJX74^ and in ORF1^M^ compared with ORF1^D^/ORF1^Glu^ were labelled in grey. **Figure S4.** Graphic genotype of the two key recombinants R-2 and R-3. White and black boxes indicated chromosomal segments from HJX74 and *O. glumaepatula*, respectively. Black triangle represented 288-bp insertion in *ORF5*^*Glu*^ promoter region compared to that of *ORF5*^*HJX74*^. **Figure S5.** Expression analysis of *S23-ORF1*. The expression of *ORF1* was analyzed in developing anthers from sporogenous cell stage to tricellular pollen stage and in other tissues including panicles, stems and leaves of HJX74, SSSL-S23 and HJX74/SSSL-S23 F_1_ plants in NSD. Spor, sporogenous cell stage. (PPT 1720 kb)

Additional file 2: **Table S1.** Primers used in this study. (DOCX 84 kb)

## Abbreviations

Indel: Insertion and deletion
NLD: Natural long-day
NSD: Natural short-day
ORF: Open reading frame
SNP: Single nucleotide polymorphism
SSR: Simple sequence repeat
SSSLs: Chromosome single-segment substitution lines
