# Supplementary figures and images for: Identification of S23 causing both interspecific hybrid male sterility and environment-conditioned male sterility in rice
Source: Rice (N Y). 2019 Feb 28;12:10. doi: 10.1186/s12284-019-0271-4 (PMC6395467; doi:10.1186/s12284-019-0271-4)

## Slide 1
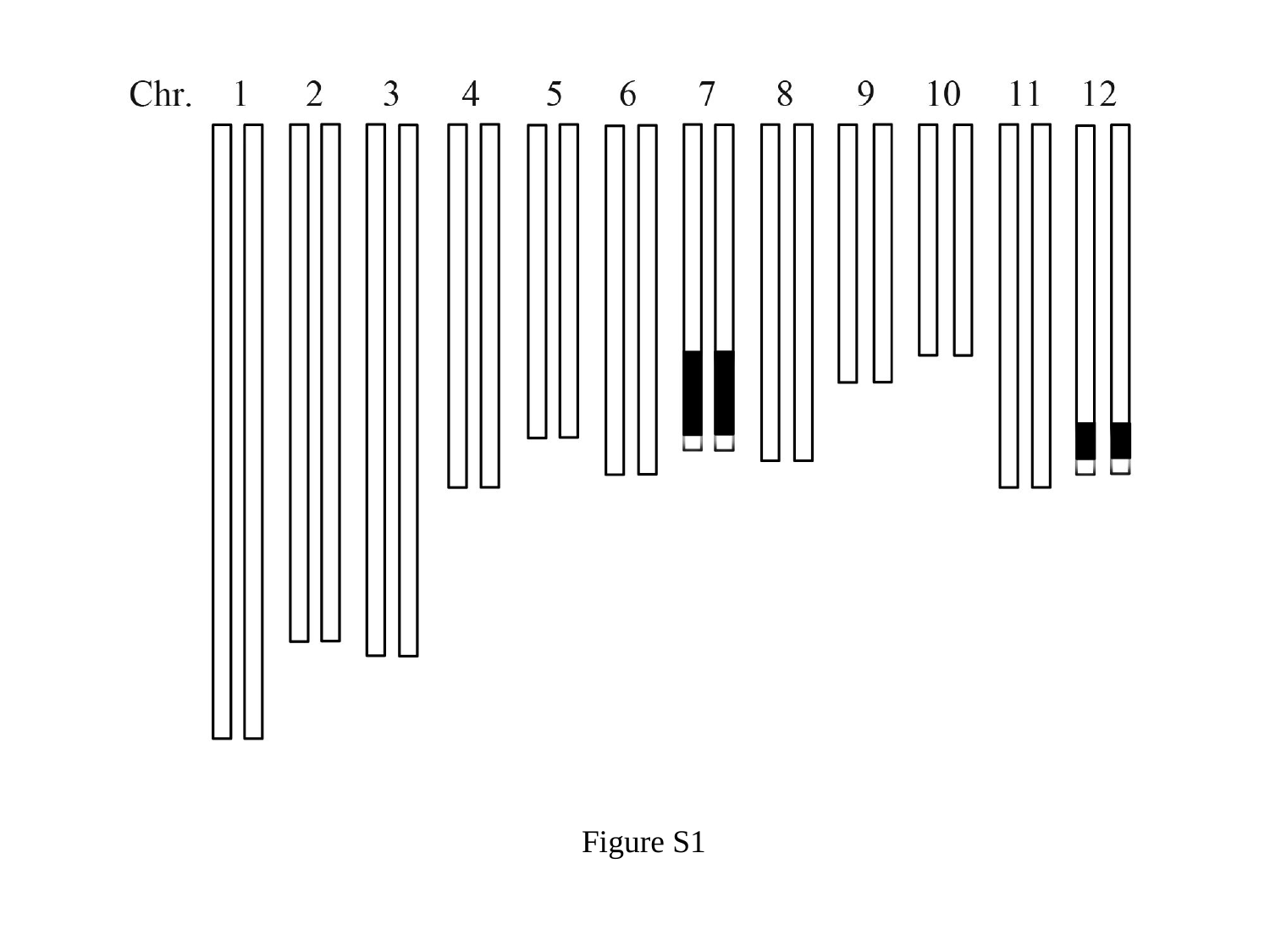

Figure S1

## Slide 2
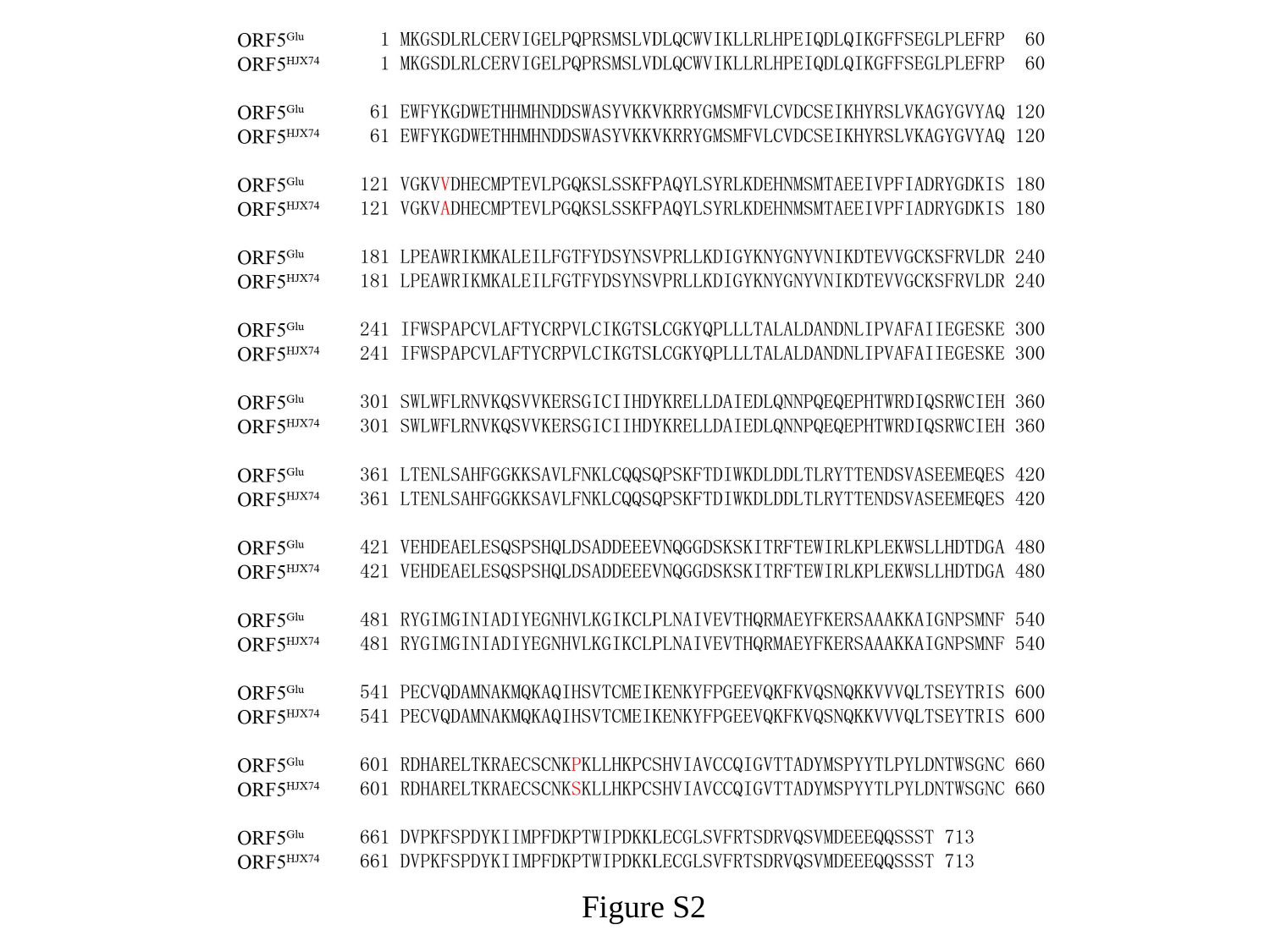

Figure S2

## Slide 3
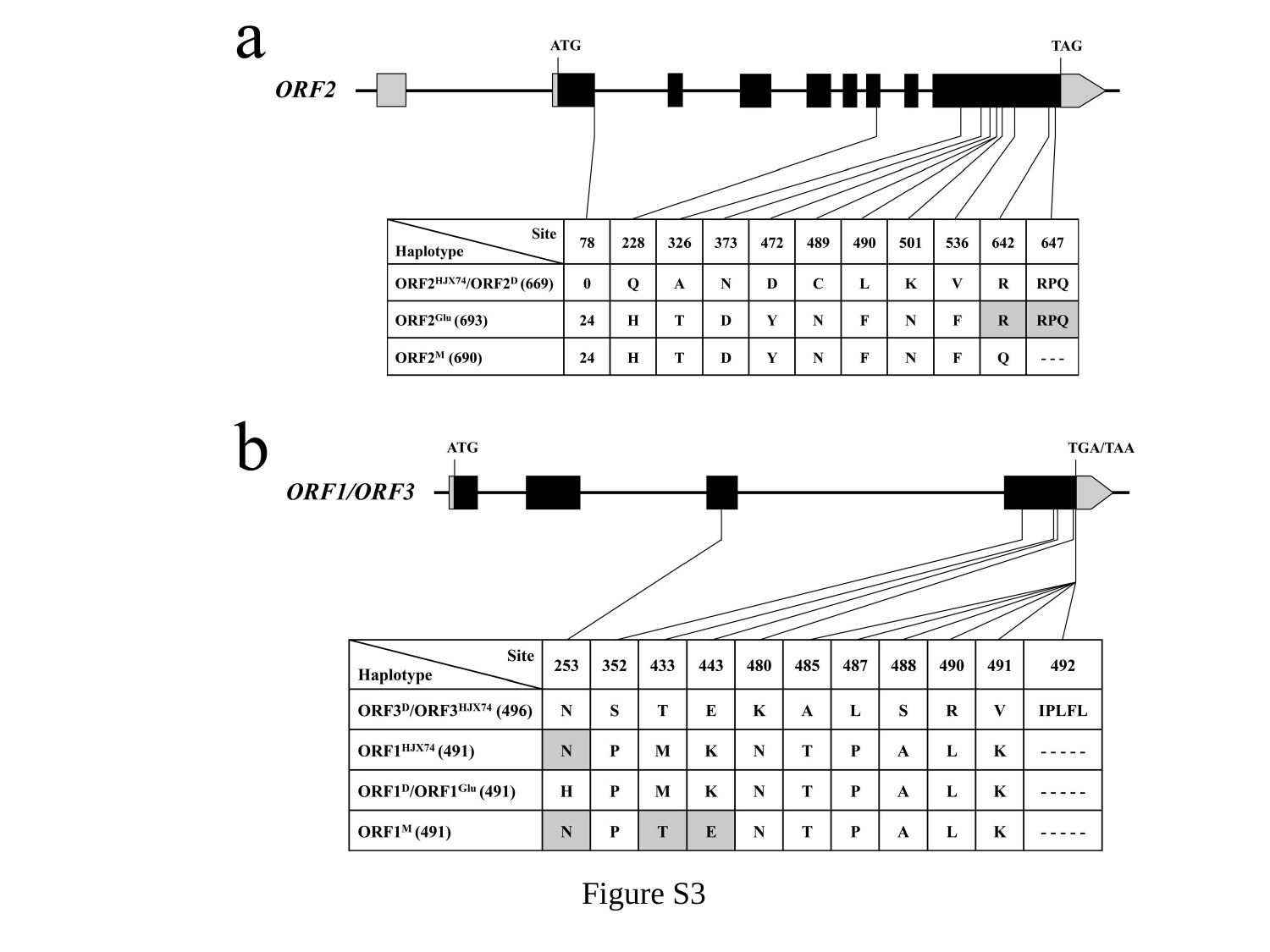

Figure S3

## Slide 4
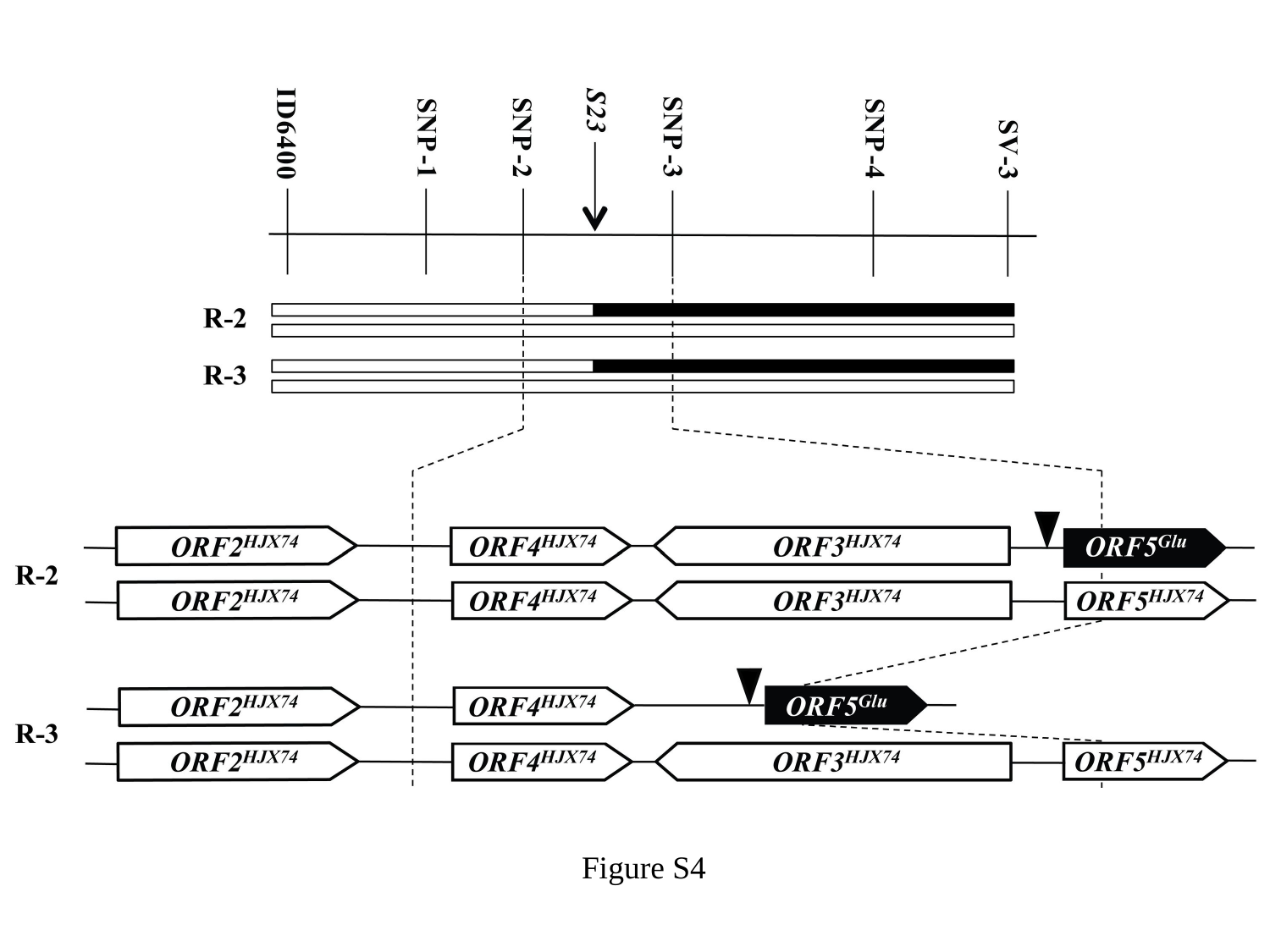

Figure S4

## Slide 5
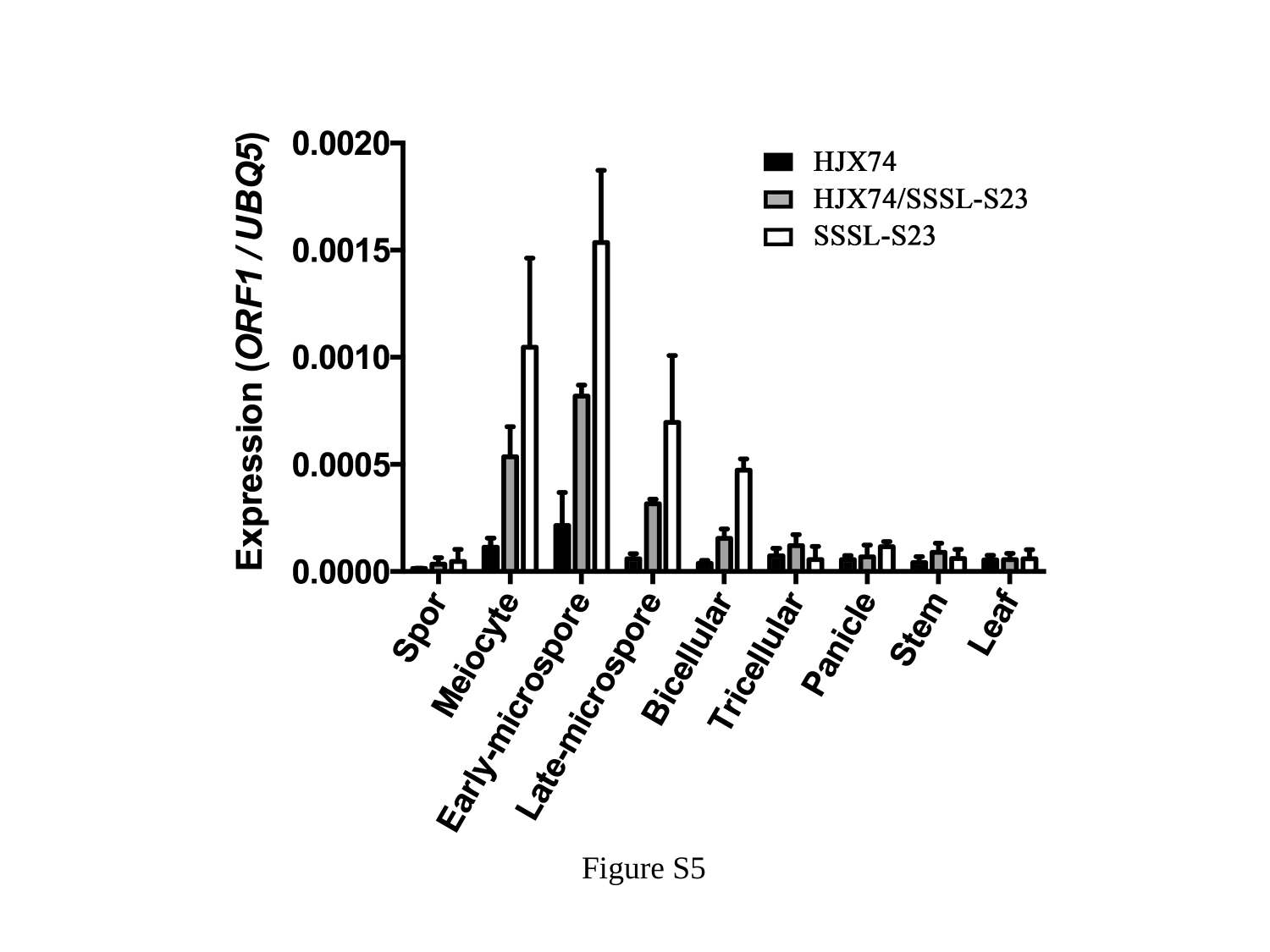

Figure S5
Figure S5

Supplement: Supplementary file 1 — Figure S1. Graphic genotype of NIL7. Black bars indicated the genomic fragments from O. glumaepatula and the other parts were from HJX74. Figure S2. Alignment of the deduced amino acid sequence of ORF5Glu and ORF5HJX74. The two amino acid substitutions were shown in red. Figure S3. Haplotype analysis of ORF2 and ORF1/3. The gene structures of ORF2 (a) and ORF1/ORF3 (b) were shown on the top and on the bottom, respectively. The black and grey blocks indicated translated regions and untranslated regions, respectively. The variable sites were shown by the vertical lines. The site numbers indicated the positions of amino acid in ORF2HJX74/ORF2D and ORF3 for the respective polymorphic sites, respectively. The haplotype numbers in parentheses indicated the length of the deduced proteins for the respective haplotypes. ORF2HJX74/ORF2D and ORF3 were functional, while ORF2Glu, ORF2M and different haplotypes of ORF1 were supposed to be non-functional. The polymorphic sites in ORF2Glu compared with ORF2M, in ORF1HJX74 and in ORF1M compared with ORF1D/ORF1Glu were labelled in grey. Figure S4. Graphic genotype of the two key recombinants R-2 and R-3. White and black boxes indicated chromosomal segments from HJX74 and O. glumaepatula, respectively. Black triangle represented 288-bp insertion in ORF5Glu promoter region compared to that of ORF5HJX74. Figure S5. Expression analysis of S23-ORF1. The expression of ORF1 was analyzed in developing anthers from sporogenous cell stage to tricellular pollen stage and in other tissues including panicles, stems and leaves of HJX74, SSSL-S23 and HJX74/SSSL-S23 F1 plants in NSD. Spor, sporogenous cell stage. (PPT 1720 kb) [file 12284_2019_271_MOESM1_ESM.ppt]
