# Supplementary material for: Identification of S23 causing both interspecific hybrid male sterility and environment-conditioned male sterility in rice
Source: Rice (N Y). 2019 Feb 28;12:10. doi: 10.1186/s12284-019-0271-4 (PMC6395467; doi:10.1186/s12284-019-0271-4)
Supplement: Supplementary file 2 — Table S1. Primers used in this study. (DOCX 84 kb) [file 12284_2019_271_MOESM2_ESM.docx]

Table S1. Primers used in this study

| Primer name | Sequence (5’-3’) | Purpose |
| --- | --- | --- |
| RM47F | ACTCCACTCCACTCCCCAC | Segregation analysis and *S23* mapping |
| RM47R | GTCAGCAGGTCGGACGTC | Segregation analysis and *S23* mapping |
| MM3579F | CTTAACATCCCCACGGAAGA | *S23* mapping |
| MM3579R | GCAAACATTCAAGGGAGGAA | *S23* mapping |
| M22049F | GGTCACTAAATACCATGAGGATACCG | *S23* mapping |
| M22049R | AGATCGAGGCCTATCCGATCC | *S23* mapping |
| M429F | TCCCTCCAGCAATGTCTTTC | Segregation analysis and *S23* mapping |
| M429R | CCTTCATCTTGCTTTCCACC | Segregation analysis and *S23* mapping |
| MM3659F | CGATTTTGCCAGATGAGGAT | *S23* mapping |
| MM3659R | GCCACAAACATAGGACAGCA | *S23* mapping |
| RID-312F | GCCAGCTCTGAATCCCATTAT | *S23* mapping |
| RID-312R | GAGCACTCGATGAGTAGCAAGA | *S23* mapping |
| RID-326F | CACACGAACAAAGTCAACCAAT | *S23* mapping |
| RID-326R | CTTTGCAGAGTGTCCGTCAT | *S23* mapping |
| InDel-5F | TGAATGTCCGGTTCACGTCACC | *S23* mapping |
| InDel-5R | ATGGACGTCTGTGATGTATCCAC | *S23* mapping |
| M22060F | CGAACCGCCTTCTCCACTGC | Segregation analysis and *S23* mapping |
| M22060R | TGTTGGTGTCCCTCTCCTAATTCTCG | Segregation analysis and *S23* mapping |
| SNP-1F | GAAGCTTAGGAACAAAGGGCGTG | *S23* mapping |
| SNP-1R | TTAGATCACGGTGGTTTGCATC | *S23* mapping |
| SNP-2F | TTGATACTGCCATAATGCCATTG | *S23* mapping |
| SNP-2R | GCCGTGGGAGCAAAGTACAACG | *S23* mapping |
| SNP-3F | TTGCTTAGGTACGAATACTGAAGC | *S23* mapping |
| SNP-3R | CATCATTATGCATATGGTGTGTC | *S23* mapping |
| SNP-4F | GTATGCTTCCAACAACTATATAC | *S23* mapping |
| SNP-4R | TGGTTGCAAATGAAATACTCTC | *S23* mapping |
| SV-3F | TCTGTAATATATCATTATGACTGC | *S23* mapping |
| SV-3R | TACTACGATATGACAATAAATCTG | *S23* mapping |
| RID-356F | CTTCCTCCTCCGTCAGGTCA | *S23* mapping |
| RID-356R | ATTCCGAGAACAACCGAGAGAG | *S23* mapping |
| RID-368F | TGCGTTATTGACTTGTCTCGAT | *S23* mapping |
| RID-368R | TGTAGACGGTATAACGCAGCAT | *S23* mapping |
| PSM147F | AATCATGGCGGATAGGTGAG | *S23* mapping |
| PSM147R | CGTGGAACAGAAAGAAAGGG | *S23* mapping |
| M22076F | TGGCGACGATAGTGGAGGAGACG | *S23* mapping |
| M22076R | CACCGGCAGCAGTCTCTGTTCG | *S23* mapping |
| PSM148F | ATGCGCACTATTTCAGGCTT | Segregation analysis and *S23* mapping |
| PSM148R | ATCCCGGAGACTTCACACAT | Segregation analysis and S23 mapping |
| M22172F | AAATCTCGCGTTCCCATTTGC | Segregation analysis and S23 mapping |
| M22172R | TAAGGCGAAGGAAGGATTGATCG | Segregation analysis and S23 mapping |
| RM17F | TGCCCTGTTATTTTCTTCTCTC | Segregation analysis |
| RM17R | GGTGATCCTTTCCCATTTCA | Segregation analysis |
| MM2968F | GCGTTGATTGATGATGATGG | Segregation analysis |
| MM2968R | CGACCCGACGTAAAAGAGAA | Segregation analysis |
| ORF4-5RACE | GCTGACAAAGCTTCTTGAACAGCATCAT | 5’RACE for ORF4 |
| ORF4-3RACE | GAGCGATCTCATATCTGCATCATACATG | 3’RACE for ORF4 |
| ORF5-5RACE | CTTCTTGCCACCAAAATGTGCAGATAAG | 5’RACE for ORF5 |
| ORF5-3RACE | ATCTGGTATCTGCATTATACATGACTAC | 3’RACE for ORF5 |
| ORF1-F | AATAGCCGTGCTGTGGCTGCGTG | qRT-PCR |
| ORF1-R | CTTGACTTACCACGTCCAACCAG | qRT-PCR |
| ORF2-F | CAAGCCCTGATTAGTGAGGCGAAG | qRT-PCR |
| ORF2-R | AATTAGTAACATCGCCAACCAG | qRT-PCR |
| ORF3-F | ATTTCTCCAATTATGTCCATCCAC | qRT-PCR |
| ORF3-R | GTTAGAGCGTCGGAGCAGACGC | qRT-PCR |
| ORF4-F | ACCACGGTTAATGTCATTGCTGG | qRT-PCR |
| ORF4-R | CTGAACCTAAGTACTGCCAACTG | qRT-PCR |
| ORF5-F | TCAAGGACACAGAGGTTGTAGGC | qRT-PCR |
| ORF5-R | ACTGGAATCAAGTTATCATTAGC | qRT-PCR |
| UBQ5-F | ACCACTTCGACCGCCACTACT | qRT-PCR |
| UBQ5-R | ACGCCTAAGCCTGCTGGTT | qRT-PCR |
